# Blood–Brain Barrier Breakdown: An Emerging Biomarker of Cognitive Impairment in Normal Aging and Dementia

**DOI:** 10.3389/fnins.2021.688090

**Published:** 2021-08-19

**Authors:** Basharat Hussain, Cheng Fang, Junlei Chang

**Affiliations:** ^1^Shenzhen Key Laboratory of Biomimetic Materials and Cellular Immunomodulation, Institute of Biomedicine and Biotechnology, Shenzhen Institute of Advanced Technology, Chinese Academy of Sciences, Shenzhen, China; ^2^University of Chinese Academy of Sciences, Beijing, China

**Keywords:** blood-brain barrier, biomarkers, cognitive impairment, aging, dementia

## Abstract

The blood–brain barrier (BBB) plays a vital role in maintaining the specialized microenvironment of the neural tissue. It separates the peripheral circulatory system from the brain parenchyma while facilitating communication. Alterations in the distinct physiological properties of the BBB lead to BBB breakdown associated with normal aging and various neurodegenerative diseases. In this review, we first briefly discuss the aging process, then review the phenotypes and mechanisms of BBB breakdown associated with normal aging that further cause neurodegeneration and cognitive impairments. We also summarize dementia such as Alzheimer's disease (AD) and vascular dementia (VaD) and subsequently discuss the phenotypes and mechanisms of BBB disruption in dementia correlated with cognition decline. Overlaps between AD and VaD are also discussed. Techniques that could identify biomarkers associated with BBB breakdown are briefly summarized. Finally, we concluded that BBB breakdown could be used as an emerging biomarker to assist to diagnose cognitive impairment associated with normal aging and dementia.

## Introduction

The central nervous system (CNS) comprises the brain and spinal cord that control all the essential functions of the body. The distinctive physiological and anatomical structure of the brain and spinal cord makes the CNS a largely immune-privileged organ (Engelhardt and Coisne, 2011; Ransohoff and Engelhardt, 2012). Blood vessels are essential to transport oxygen and nutrients, remove CO_2_ and other waste products, and, thus, maintain homeostasis in the body. Blood vessels that vascularize the CNS acquire specific anatomical and functional characteristics that collectively form the blood–brain barrier (BBB) (Obermeier et al., 2013; Zhao et al., 2015).

**Figure 1:**
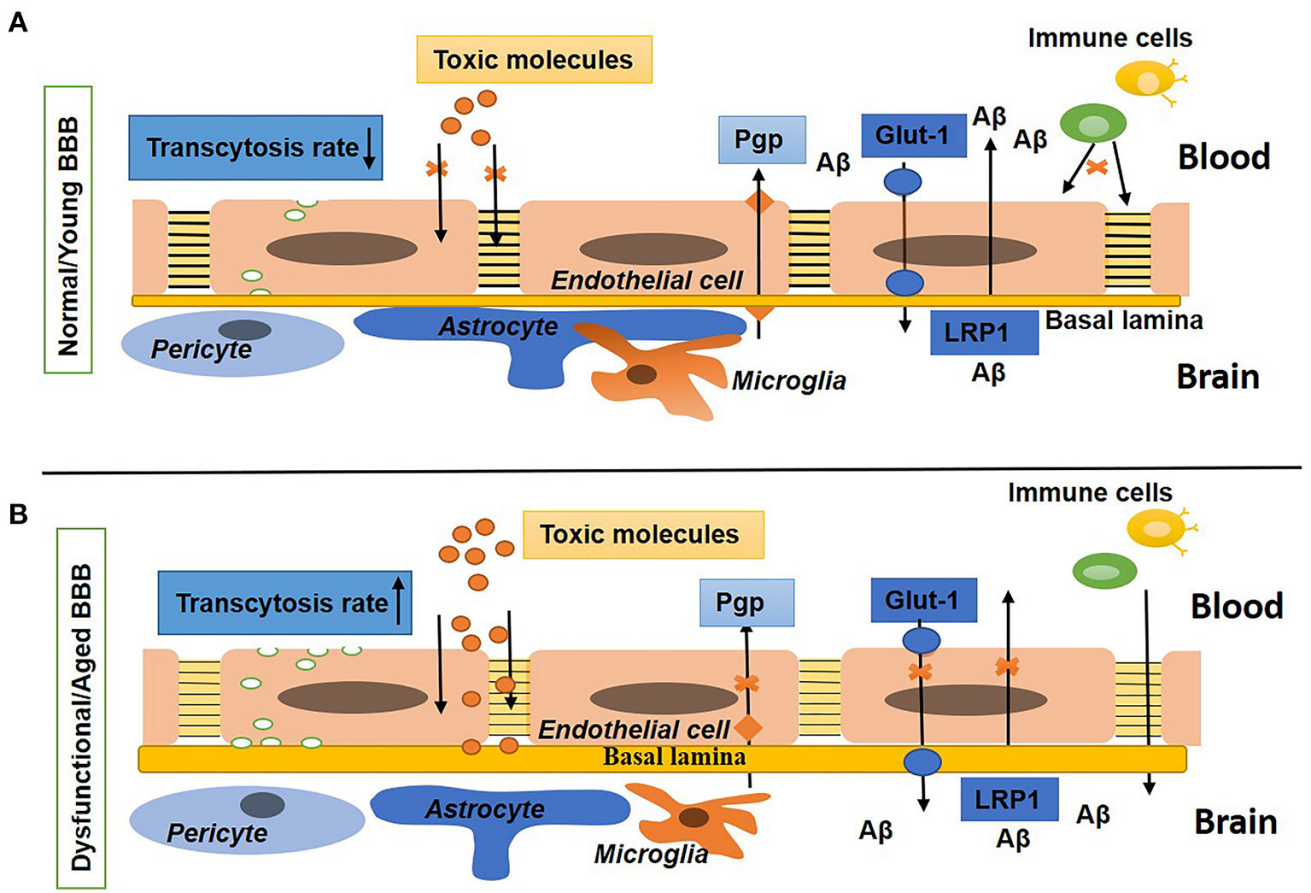
Schematic diagram shows the normal/young blood–brain barrier (BBB) and dysfunctional/aged BBB. **(A)** Shows BBB in a young or normal state with tight and adherens junctions, a low rate of transcytosis, no diffusion of toxins, the presence of influx (Glut-1) and efflux (P-gp) transporters, and a low expression of leukocytes adhesion molecules (LAMs). The basal lamina is thin and surrounded by pericytes, astrocyte endfeet, and microglia. **(B)** Shows BBB in an aged or disease state with a high rate of transcytosis and diffusion of toxins, repression in influx and efflux transporters, upregulated expression of LAMs, and increased density of the extracellular matrix (ECM). Pericytes, astrocytes, and microglia are not associated with the basal lamina.

At the cellular level, the BBB is developed by continuous non-fenestrated endothelial cells (ECs) encompassed by pericytes, smooth muscle cells, astrocytes, microglia, oligodendroglia, and neurons that are altogether called the neurovascular unit (NVU) (Zlokovic, 2011; Blanchette and Daneman, 2015; Chow and Gu, 2015). At the molecular level, the BBB ECs are compacted by claudins, occludins, and ZO-1 [tight junction (TJ) proteins] and junction adhesion molecules (JAM) proteins to restrict the paracellular and transcellular diffusion of molecules in the CNS. In addition, the BBB ECs mediate influx transporters to select metabolite uptake from the blood and efflux transporters to remove toxins and waste products from the brain into the blood. In BBB ECs, leukocyte adhesion molecules (LAMs) express very low to suppress immune surveillance in the brain (Quaegebeur et al., 2011; Engelhardt and Ransohoff, 2012; Chow and Gu, 2015; Xiao et al., 2020). Thus, the BBB confines the access of neurotoxic compounds, blood cells, and pathogens to the brain (Winkler et al., 2011). In addition, the BBB sustains the homeostasis of the brain through tight regulation of the transport of molecules between the brain parenchyma and peripheral circulation (Abbott, 2013). Figure 1A shows the normal BBB.

Hence, the BBB is a fundamental and crucial element of normal and healthy brain function. Any impairment in the cellular or molecular components causes BBB breakdown that results in BBB dysfunction. Aging is one of several factors involved in the breaking of the BBB and was first observed in aged patients reported in the 1970s (Tibbling et al., 1977). In dysfunctional BBBs, the possibility of permeability increases; thus, toxic and blood-borne inflammatory substances that infiltrate the brain could change the biochemical microenvironment of the neurons, thus leading to neurodegenerative diseases and dementia (Abbott et al., 2010; Zeevi et al., 2010; Zlokovic, 2011; Rosenberg, 2014; Sweeney et al., 2018b). It has been reported that BBB disruption in aged people is strongly related to Alzheimer's disease (AD) and cognitive impairment (Farrall and Wardlaw, 2009; Van De Haar et al., 2016; Skillbäck et al., 2017; Zenaro et al., 2017; Sweeney et al., 2018b; Nation et al., 2019). Figure 1B shows the impaired BBB.

In this review, we first briefly discuss the aging process, and then review the phenotypes and mechanisms of BBB breakdown associated with normal aging that further cause neurodegeneration and cognitive impairments. We also summarize dementia such as AD and vascular dementia (VaD); then, we discuss the phenotypes and mechanisms of BBB disruption in dementia correlated with cognition decline. Subsequently, we also discuss the overlap between AD and VaD. Furthermore, we mention biomarkers associated with BBB breakdown during aging and dementia; additionally, we also briefly discuss various techniques to identify BBB biomarkers. Finally, we conclude that BBB breakdown could be used as a novel biomarker to diagnose cognitive impairment associated with normal aging and dementia.

## BBB Breakdown in Normal Aging

The universal process in an organism leads to the cumulation of biological variations responsible for progressively diminishing bodily functions over time, which is known as aging (Kritsilis et al., 2018). Because of the advancement in medicine and the living standard of humans, life expectancy has doubled worldwide (Aw et al., 2007). Aged people are estimated to make up approximately 20% of the world population in the next 50 years (Ellison et al., 2015). In terms of the brain and the BBB, normal aging can be defined as a retrogression in the activities of the body with no cognitive ailment and dementia. Although ailments do not occur in this case, the frequency of age-related diseases increases with the aging process. Alzheimer's, cardiovascular, Parkinson's disease, stroke, and various other neurological diseases commonly occur in aged people (Erdo et al., 2017). A recent study demonstrated that BBB breakdown could be considered a biomarker for the normal aging process (Verheggen et al., 2020). Furthermore, BBB breakdown also impairs the influx of nutrients (glucose) and oxygen and efflux of waste products, which may cause hypoxia-associated inflammation (Elahy et al., 2015; Raja et al., 2018). Subsequently, age-related BBB pathology makes the brain more susceptible to neuronal impairment and even causes neurodegeneration (Levit et al., 2020; Banks et al., 2021). It has been reported that aged people with prior cognitive impairment were more vulnerable to BBB disruption than people with no cognitive dysfunction of the same age; hence, BBB disruption can be considered an early biomarker related to declines in human cognition (Nation et al., 2019). All these studies show that the way alterations in BBB components progress with time might be an interesting research topic to explore in association with the normal aging brain.

### Phenotypes of BBB Breakdown in Normal Aging

During aging, various changes occur in the structure and function of brain vasculature. In the aged brain, the BBB becomes broken; hence, the permeability of the BBB elevates (Villeda et al., 2011; Hyman et al., 2012) and declines in the cerebral blood flow (CBF) occur (Tarumi and Zhang, 2018). The potency of neovascularization diminishes (Rivard et al., 2000; Gao et al., 2009) and the density of capillary of brain vasculature reduces with age (Reeson et al., 2018). It has been observed that, during aging, BBB breakdown is the first incident that starts in the hippocampus, which may lead to declines in cognition (Montagne et al., 2015). In normal aging, the main changes that are strongly correlated to BBB breakdown are presented in Table 1.

**Table 1:** Changes associated with blood–brain barrier (BBB) breakdown in normal aging, Alzheimer's disease (AD), and vascular dementia (VaD).

**BBB elements**	**Characteristics**	**Aging**	**References**	**AD**	**References**	**VaD**	**References**
ECs	• Endothelium degeneration, Mitochondrial content decrease, pinocytotic vesicle increase • Microvessel density decrease	Yes	(Bell and Zlokovic, 2009; Grinberg and Thal, 2010; Richardson et al., 2012; Rouhl et al., 2012; Sagare et al., 2012)	Yes	(Salmina et al., 2010; Villar-Vesga et al., 2020; Chacón-Quintero et al., 2021; González-Molina et al., 2021)	Yes	(Wardlaw et al., 2003; Zhang et al., 2014; Rajani et al., 2018; Wang et al., 2018; Tayler et al., 2021; Zhu et al., 2021)
Extracellular components	• Increase (accumulation)	Yes	(Brown and Thore, 2011)	Yes	(Zlokovic, 2011; Hawkes et al., 2013; Morris et al., 2014; Howe et al., 2020)	Yes	(Ueno et al., 2002; Rosenberg, 2017)
Basal lamina	• Thickness increase	Yes	(Grinberg and Thal, 2010; Richardson et al., 2012; Rouhl et al., 2012)	Yes	(Zlokovic, 2011; Morris et al., 2014)	Yes	(Ueno et al., 2002; Iadecola, 2013; Rosenberg, 2017)
Pericytes	• Pericytes number decrease • PDGFRβ in CSF increase	Yes	(Bell et al., 2010; Montagne et al., 2015; Sagare et al., 2015; Duncombe et al., 2017; Erdo et al., 2017; Goodall et al., 2018; Nation et al., 2019)	Yes	(Sengillo et al., 2013; Halliday et al., 2016; Montagne et al., 2018; Miners et al., 2019; Uemura et al., 2020)	Yes	(Iadecola, 2013; Montagne et al., 2018; Yang et al., 2018; Uemura et al., 2020)
Astrocytes	• Vascular coverage reduction • GFAP upregulation • AQP4 downregulation	Yes	(Middeldorp and Hol, 2011; Duncombe et al., 2017; Goodall et al., 2018; Heithoff et al., 2021)	Yes	(Abbott et al., 2006; Yang Y. et al., 2011; Kimbrough et al., 2015; Ahmad et al., 2019)	Yes	(Wardlaw et al., 2003; Iadecola, 2013; Saggu et al., 2016; Price et al., 2018; Wang et al., 2018; Tayler et al., 2021)
Microglia	• Release of neurotoxins • Changes to amoeboid morphology	Yes	(Kettenmann et al., 2011; Ronaldson and Davis, 2012)	Yes	(Zotova et al., 2011; Hansen et al., 2018; Ahmad et al., 2019; Hemonnot et al., 2019; Leng and Edison, 2020)	Yes	(Wu et al., 2016; Wang et al., 2018; Tayler et al., 2021)
Neurons	• Synaptic plasticity diminishment • Impaired long-term potentiation • Dysfunctional neurogenesis • Elevation in apoptosis • Neurodegeneration	Yes	(Buschini et al., 2011; Blau et al., 2012; Cerbai et al., 2012; Lucke-Wold et al., 2014)	Yes	(Crews and Masliah, 2010; Arendt et al., 2015; Vasic et al., 2019; Bartels et al., 2020)	Yes	(Saggu et al., 2016; Montagne et al., 2018; Wang et al., 2018; Tayler et al., 2021; Zhu et al., 2021)
Tight junctions Proteins	• *CLDN5, OCLN, ZO-1* expression decreases and BBB integrity reduction, BBB permeability increase	Yes	(Bake et al., 2009; Wang et al., 2011; Lassman et al., 2012; Elahy et al., 2015)	Yes	(Biron et al., 2011; Cuevas et al., 2019; Yamazaki et al., 2019)	Yes	(Wang et al., 2018; Yang et al., 2018)
Transporter dysfunctions	• Influx transporter: Glut1 expression decrease, glucose uptake reduction • Efflux Transporter: LRP-1 (human) and P-gp expression decrease (mouse)	Yes	(van Assema et al., 2012; Ding et al., 2013; Jiang et al., 2013; Chiu et al., 2015; Ramanathan et al., 2015; Hoffman et al., 2017; Patching, 2017; Sweeney et al., 2019a)	Yes	(Owen et al., 2010; Jaeger et al., 2011; Ding et al., 2013; Chiu et al., 2015; Ramanathan et al., 2015; Winkler et al., 2015; Halliday et al., 2016; Patching, 2017; Yu et al., 2020; Kyrtata et al., 2021)	Yes	(Hase et al., 2019)
Circulating factors	• ASM (acid sphingomyelinase), sphingomyelin phosphodiesterase 1 (Smpd1) upregulation	Yes	(Park et al., 2018; Wangb et al., 2019)	*		*	
Other factors	• SIRT1 expression decrease	Yes	(Chang and Guarente, 2014; Imai and Guarente, 2014; Stamatovic et al., 2019)	*		*	

“*” shows no obvious research studies are found related to AD and VaD.

It has been reported that, in aging, the brain endothelium becomes progressively dysfunctional, which is correlated with aberrant changes in the BBB (Cai W. et al., 2017; Edwards et al., 2019). The extracellular matrix (ECM) of the basal membrane or basal lamina covers the brain endothelium and is considered uniform and thin. In normal aging, the thickness of the ECM increases with the increase in collagen IV and argin but decreases in laminin concentrations (Candiello et al., 2010). Although the ECM has a role in maintaining BBB integrity by inducing TJ (occludin) protein expression, changes in the ECM cause BBB disruption, and thus result in increased BBB permeability (Hawkins and Davis, 2005; Candiello et al., 2010; Sanchez-Covarrubias et al., 2014).

In the CNS BBB, ECs associated with pericytes, astrocytes, neurons, and glial cells that develop and maintain their specific phenotype led to BBB integrity (Erickson and Banks, 2018). However, with aging, this association caused BBB breakdown. During aging, physiological ultrastructure changes have been reported in pericytes, such as an increase in mitochondria size (Hicks et al., 1983), vesicular and lipofuscin-like inclusions (Rascher and Wolburg, 2002), and foamy transformations (Sturrock, 1980). In addition, the protrusions on the basal lamina or the ECM membrane of the microvessels have been observed to result in the degeneration of pericytes (Ueno et al., 1998). A loss of pericytes has also been reported in aging mice, rats, and the human brain (Stewart et al., 1987; De Jong et al., 1990; Bell et al., 2010; Duncombe et al., 2017; Goodall et al., 2018) but some studies observed that the number of pericytes increases in aged rat brains (Heinsen and Heinsen, 1983; Peinado et al., 1998). However, no change was observed in the number of pericytes in aged monkey brains (Peters et al., 1991).

Platelet-derived growth factor receptor beta (PDGFRβ) maintains the phenotype of pericytes in the brains of aged mice with *PDGFR*β^+^^/−^, which shows that the loss of pericytes leads to BBB breakdown and increased BBB permeability (Bell et al., 2010). It has been reported, in the aged human brain, the level of soluble PDGFRβ in cerebrospinal fluid (CSF) increases, showing damage to the pericyte associated with BBB disruption (Montagne et al., 2015; Sagare et al., 2015; Nation et al., 2019). In addition, it has been reported that, in APOE4 carriers, the elevated PDGFRβ in the CSF may be used as a biomarker of cognitive impairment (Montagne et al., 2020).

The endfeet of astrocytes that ensheath the pericytes have a contribution to BBB development and maintenance. With the age, vascular coverage and aquaporin-4 (AQP4) expression of astrocyte endfeet are reduced whereas glial fibrillary acidic protein (GFAP) expression and endfeet sizes are increased (Middeldorp and Hol, 2011; Duncombe et al., 2017; Goodall et al., 2018), leading to increase in reactive astrogliosis.

Microglia are distributed ubiquitously in the CNS and activated during aging and pathology (Kettenmann et al., 2011; Kofler and Wiley, 2011; Harry, 2013; Sanchez-Covarrubias et al., 2014). Microglia have a ramified structure in the resting state, but when activated, this structure changes into an amoeboid morphology during aging or a pathophysiological state (Kettenmann et al., 2011). During aging or stress, the activated microglia produce tumor necrosis factor-α (TNF-α), proteases, nitric oxide (NO), and peroxide (Ronaldson and Davis, 2012), which are associated with an alteration in the TJ protein. This alteration induces BBB leakage (Huber et al., 2006), which, in turn, leads to cell injury and neurodegeneration (Ronaldson and Davis, 2012).

Studies have shown that neurons directly connect with brain ECs and astrocytes (Ben-Menachem et al., 1982; Cohen et al., 1996, 1997; Tong and Hamel, 1999; Vaucher et al., 2000; Sanchez-Covarrubias et al., 2014). Impairment in this association results in BBB breakdown and leads to an increase in BBB permeability to albumin (Berezowski et al., 2004). Figure 1 shows the difference between young or normal BBB and aged or dysfunctional BBB.

### Mechanisms of BBB Breakdown During Normal Aging

**Figure 2:**
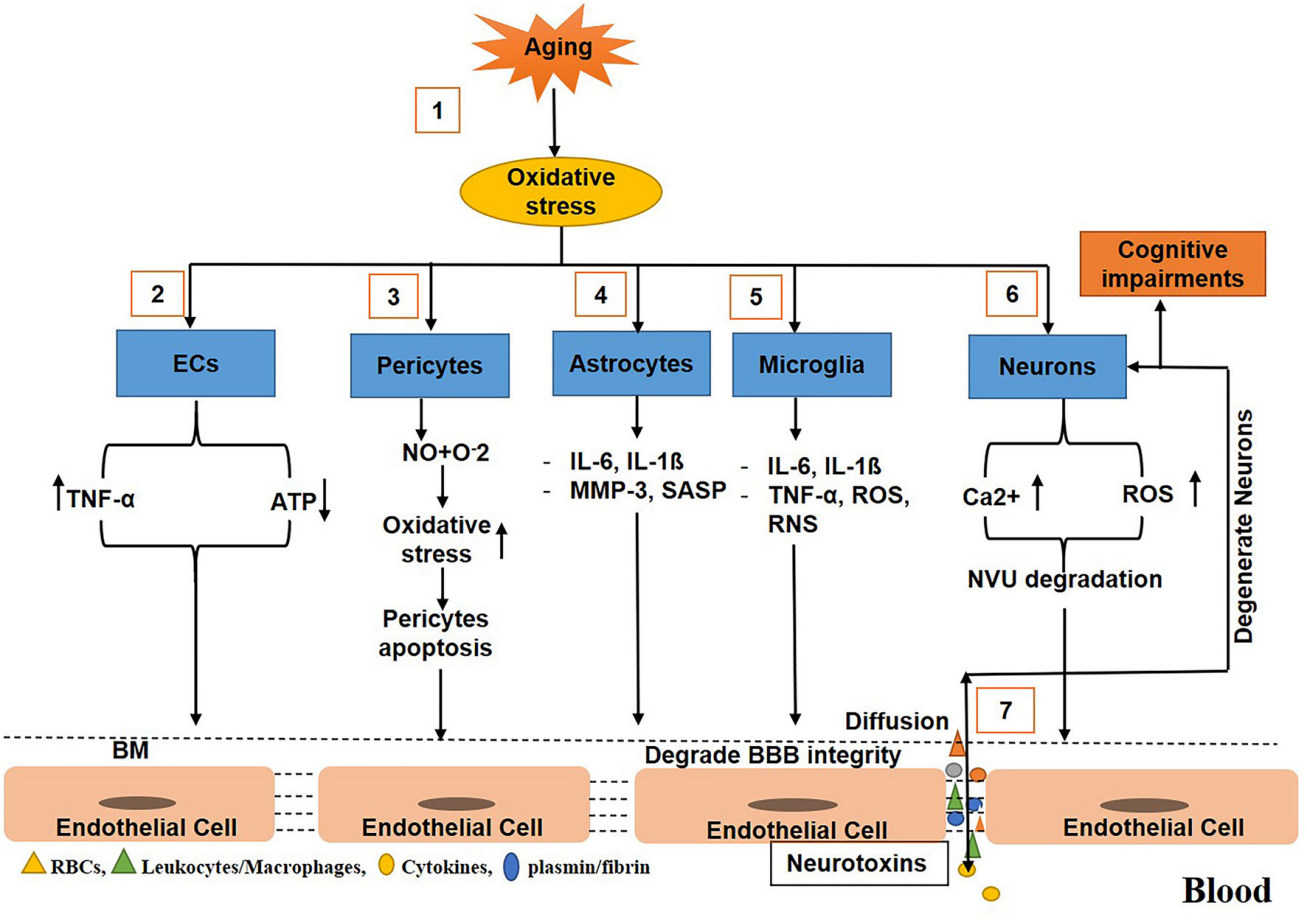
Schematic diagram shows the mechanisms of blood–brain barrier (BBB) breakdown in normal aging. (1) Oxidative stress increases with age. (2) Oxidative stress triggers endothelial cells ECs to release tumor necrosis factor-α (TNF-α) and consume more ATP. (3) Oxidative stress induces pericytes to release nitric oxides that react with reactive oxygen to further upregulate the oxidative stress causing pericytes apoptosis associated with loss in BBB integrity. (4) During aging, oxidative stress stimulates and activates astrocytes to release cytokines and chemokines that degrade the basement membrane and tight junction leading to BBB impairment. (5) Oxidative stress also activates the microglia to secrete cytokines, chemokines, reactive oxygen, and nitrogen species, causing degradation in BBB integrity. (6) Oxidative stress also induces neurons to release reactive oxygen species (ROS) and calcium ions accumulation that cause to degrade the neurovascular unit (NVU). (7) Toxins freely diffuse to and from the brain, causing neurodegeneration and decline in cognition.

During aging, various mechanisms cause BBB breakdown and increase BBB permeability. For example, in aging, oxidative stress induces ECs to produce TNF-α that cause the degradation of the basement membrane, and TJs (Occludin, Zonula occludins-1), which, in turn, results in BBB disruption and an increase in BBB permeability (Donato et al., 2007; Bake et al., 2009; Lee et al., 2012; Elahy et al., 2015; Cai W. et al., 2017). In addition, the activity of caspase 3/7 in the aged brain increases, which causes the suppression of cell viability and the upregulation of apoptosis in pericytes (Schultz et al., 2018), resulting in the reduction of the number of pericytes in the BBB (Bell et al., 2010). The senile pericytes produce NO and react with O_2_, causing increased oxidative stress and compromised BBB integrity (Hughes et al., 2006; Sweeney et al., 2016; Cai W. et al., 2017). Similarly, in aging, oxidative stress enhances astrocytes to upregulate the expression of cytokines and chemokines, such as matrix metalloproteinase 3 (MMP3) and p16INK4A [senescence-associated secretory phenotype (SASP)], that induce BBB disruption, neuroinflammation, and cognitive impairments (Simpson et al., 2010; Salminen et al., 2011; Cai Z. et al., 2017; Bussian et al., 2018). In aging, oxidative stress also activates the microglia to release cytokines, chemokines IL-6, IL1ß, and TNF-α, which results in the elevation of reactive oxygen and nitrogen species; this ultimately causes the breakdown of the BBB (Gredilla et al., 2010; Choi et al., 2014; Fivenson et al., 2017). With age, oxidative stress causes the production of reactive oxygen species (ROS) to elevate in the CNS, but the capability of neurons to clear ROS decreases, resulting in neurodegeneration (Nicholls and Budd, 2000; Mattson and Magnus, 2006). Furthermore, with age, calcium dysregulation in neurons occurs, which represses calcium-binding proteins correlated with the elevation of ROS. This results in BBB degradation and neuronal loss (He et al., 1997) as shown (Figure 2).

**Figure 3:**
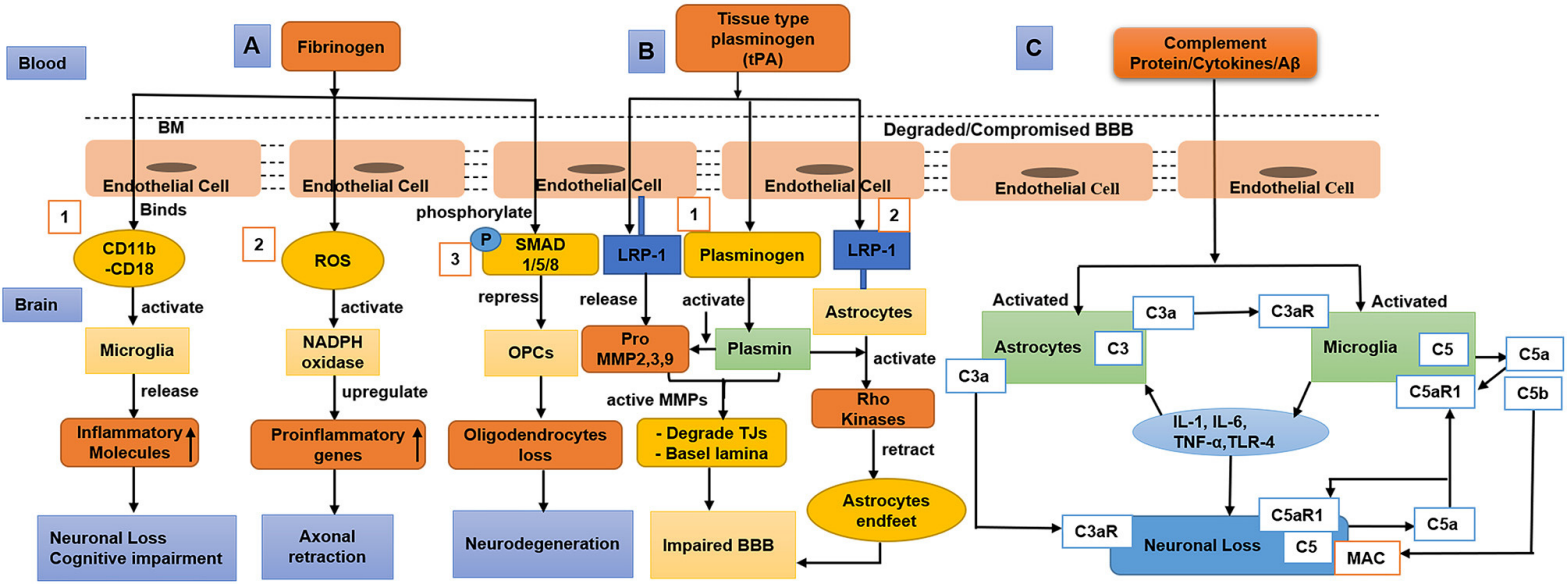
Diagram that shows fibrinogen, tissue-type plasminogen activator (tPA), plasmin, and complement proteins/cytokines/Aβ cross compromised the blood–brain barrier (BBB), causing neuronal loss and decline in cognition. **(A)** (1) Fibrinogen activates the microglia *via* CD11b/CD18, which releases inflammatory/toxic molecules causing neurodegeneration and cognitive impairment. (2) Fibrinogen induces reactive oxygen species (ROS), activates nicotinamide adenine dinucleotide phosphate (NADPH oxidase), and upregulates proinflammatory genes causing axonal retraction and cognitive impairment. (3) Fibrinogen phosphorylates SMAD 1/5/8 and represses oligodendrocyte progenitor cells (OPCs) leading to oligodendrocyte loss. **(B)** (1) tPA binds with low-density lipoprotein receptor-related protein-1 (LRP-1) of endothelial cells (ECs), which secretes pro-matrix metalloproteinases (MMPs). tPA activates plasminogen into plasmin, which further activates pro-MMPs that degrade tight junctions (TJs) and basal lamina. (2) tPA also binds with the LRP-1 of astrocytes, induces plasmin mediated activation of Rho kinase, and results in astrocyte retraction leading to BBB impairment. **(C)** During aging and dementia such as Alzheimer's disease (AD), complement proteins, oxidative stress, and the aggregated amyloid-beta (Aβ) activate the astrocytes and microglia, leading to neuroinflammation. The C3aR and C5aR1 signalings on activated microglia cause the release of cytokines [IL-1, IL-6, tumor necrosis factor-α (TNF-α), and toll-like receptor 4 (TLR-4)] and result in neurodegeneration. To aggravate neuronal apoptosis, C5a as a neuronal-derived signal that interacts with C5aR1 on neurons in an autocrine way. Furthermore, C3a as an astrocytic-derived signal binds to C3aR on neurons to exacerbate neuronal morphology. During neuroinflammation, microglia-derived C5b binds with the membrane attack complex (MAC) that enhances neuronal loss.

Once the BBB integrity becomes compromised, blood-derived proteins such as fibrinogen and plasminogen cross the BBB, and the pro-inflammatory fibrin aggregates in the brain (Cortes-Canteli et al., 2015). A study using a mouse model showed that accumulated fibrin bind with CD11b/CD18 and activate microglia, which then triggers a decline in cognition (Merlini et al., 2019). Accumulated fibrin in brain also induce increased ROS level and activates nicotinamide adenine dinucleotide phosphate (NADPH oxidase), which upregulates pro-inflammatory gene expression and causes damage to neuronal axons (Ryu et al., 2018; Merlini et al., 2019). In addition, fibrinogen phosphorylates Smad 1/5/8 represses oligodendrocyte progenitor cells (OPCs) (Ryu et al., 2015). Furthermore, the complex of Aβ-fibrinogen activates microglia *via* CD11b/CD18, which inhibits the breakdown of fibrinogen and promotes neuronal degeneration (Cortes-Canteli et al., 2010; Zhao et al., 2017) as shown (Figure 3A).

The tissue-type plasminogen activator (tPA) binds and activates low-density lipoprotein receptor-related protein-1 (LRP-1) on ECs. In run, these ECs produce pro-MMPs (MMP-2, MMP-3, and MMP-9) (Wang et al., 2003; Cheng et al., 2006; Suzuki et al., 2009). Subsequently, tPA converts the surface-bound inactive plasminogen (Plg) into active plasmin (Plm) (Doeuvre et al., 2010; Yepes et al., 2021). Plasmin, in turn, activates the MMPs, leading to the degradation of TJs and basal lamina (Mazzieri et al., 1997; Ramos-DeSimone et al., 1999; Monea et al., 2002; Rosenberg and Yang, 2007; Yang Y. et al., 2011). Furthermore, tPA also binds with LRP-1 on astrocytes, which induces plasmin-mediated activation of Rho kinases and retracts the endfeet of the astrocytes from the blood vessel wall, thus resulting in BBB dysfunction (Niego et al., 2012). In addition, a study suggested that plasminogen might regulate brain inflammation during AD (Baker et al., 2018) as shown (Figure 3B).

Human brain ECs continuously produce complement regulatory proteins and components (Wu et al., 2016), which are elevated by CNS injury or infiltration into the brain when the BBB is dysfunctional. However, at a young age or in a normal state, complement proteins mostly do not cross the BBB (Hoarau et al., 2011; Veerhuis et al., 2011). Once the complement proteins cross the compromised BBB, they could alter the functions of the microglia, oligodendrocytes, and neurons (Orsini et al., 2014). Complement activation produces C3a and C5a that interact with C3aR and C5aR1, respectively, which play a significant role in the infiltration of inflammatory cells into the brain and the induction of cytokine cascades (IL-1, TNF-α, IL-6, IL-8, IL-17) subsequently leading to neurodegeneration (Jacob and Alexander, 2014; Alexander, 2018). In AD, amyloid-beta (Aβ) activates the complement signaling by binding to C1q. Inhibition of the C5/C5aR1 pathway was also reported to be a protective therapeutic target in AD (Fonseca et al., 2009) as shown (Figure 3C).

## BBB Breakdown in Dementia (Including AD and Vascular Dementia)

Dementia is a group of conditions or disorders that affect the functions of the brain. It is a progressive neurological disease associated with impairments in cognition and deterioration of the everyday life activities of an affected individual (Mills et al., 2007; Kirshner, 2009). In dementia, BBB breakdown and cerebral hypoperfusion cause brain damage and a decline in cognition (Nation et al., 2019; Tayler et al., 2021). Dementia is a considerable health complication affecting millions of people worldwide. In developed countries, AD and VaD are two significant types of dementia with a prevalence of about 4.4 and 1–2%, respectively (Ray et al., 2013), with AD being the most common type of dementia in aged people (Ballaed et al., 2011; Hyman et al., 2012). The World Alzheimer Report 2018 estimated that approximately 50 million people of the global population suffer from dementia, which can increased to 82 million in 2030 and triple to 152 million by 2050 (Patterson, 2018). Alzheimer's is considered to account for 60–70% of all dementia cases worldwide (Leng and Edison, 2020). As the BBB has vital contributions to maintaining the microenvironment of the CNS, any impairment in the cellular or molecular components of the BBB can cause various neurodegenerative diseases, including AD (Zlokovic, 2005; Erickson and Banks, 2013; Zenaro et al., 2017). After AD, VaD is the second most common type of dementia, accounting for 15% of all dementia cases worldwide (O'Brien and Thomas, 2015). Vascular dementia is a type of neurological disease with a defect in cognition caused by impairment in the vascular system, such as a reduction in CBF (Sabayan et al., 2012). Various vascular pathologies are associated with VaD, such as infarcts and white matter (WM) alterations (O'Brien and Thomas, 2015). In addition, brain hemorrhage, ischemia, and hypoxia may be the causing factors of VaD (Kirshner, 2009; Grinberg and Heinsen, 2010).

### BBB Breakdown in AD

#### Pathophysiology of AD

Aging is responsible for pathophysiological changes that aggravate neurological diseases. It causes the thickening of the wall of the blood vessel and increases blood vessel tortuosity, which may lead to BBB disruption (Rosenberg, 2012). The BBB breakdown in AD results in the accumulation of insoluble extracellular plaques of β-amyloid (Aβ) along the walls of blood vessels and causes inflammation in the NVU (Kinnecom et al., 2007; Kang et al., 2017). In neuronal cytoplasms, the accumulation of neurofibrillary tangles (NFT) of P-tau is also associated with AD (Kang et al., 2017). It has been observed that, in AD, the reduction of Aβ clearance is correlated with declines in CBF and cognitive impairment (Sagare et al., 2012). These pathological markers are associated with BBB impairment, which causes microglial activation, neuroinflammation, degeneration of neurons, and cognitive impairment (Bhaskar et al., 2010; Iadecola, 2013). As pericytes have a crucial role in the development and maintenance of BBB, their number and density decreased in the cortex and hippocampus of AD patients (Sengillo et al., 2013), subsequently leading to the upregulation of the expression of Aβ and p-tau protein (Sagare et al., 2013).

**Figure 4:**
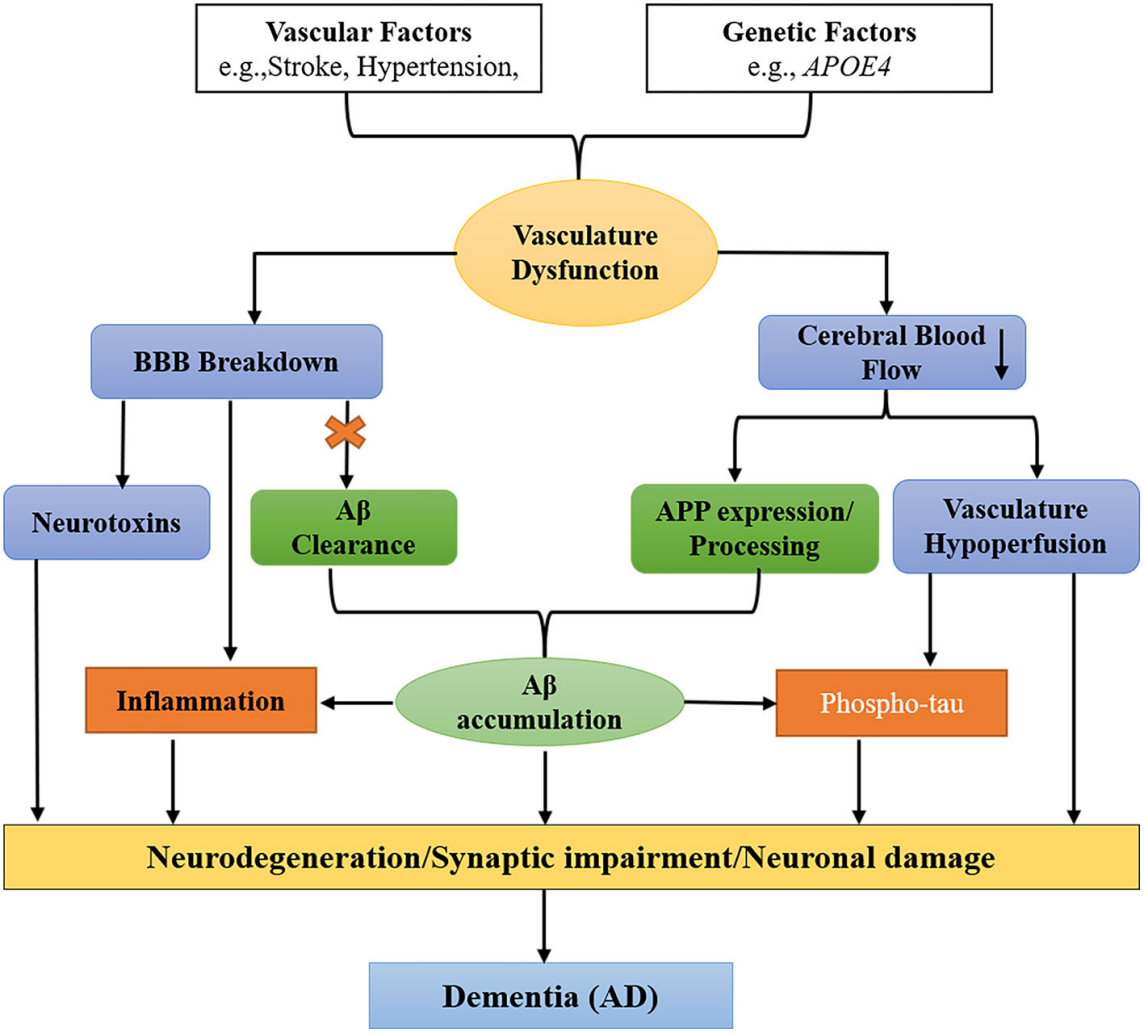
Schematic diagram shows two pathways that cause dementia [specifically Alzheimer's disease (AD)]. Vascular factors (stroke, hypertension, diabetes, etc.) and genetic factors (such as *APOE4*) cause defects in the vascular system leading to blood–brain barrier (BBB) impairment and oligemia (reduced cerebral blood flow), which finally correlate with dementia and neuronal degeneration. In the amyloid-beta (Aβ)-independent pathway (blue), vascular and genetic factors cause BBB breakdown and the secretion of neurotoxins from one side and oligemia from the other side. While in the Aβ-dependent pathway (green), the first BBB breakage impairs Aβ clearance and the amyloid precursor protein (APP), resulting in the accumulation of Aβ in the brain. Vascular hypoperfusion and Aβ phosphorylate tau (p-tau) that forms neurofibrillary tangles (NFTs). Finally, both pathways cause neurodegeneration/synaptic impairment/neuronal damage leading to dementia (specifically AD).

Vascular (stroke, hypertension, diabetes, etc.) and genetic factors (*APOE4*) are two pathways that cause BBB impairment and oligemia (reduced CBF) that result in dementia. In the Aβ-independent pathway (blue), the BBB breakdown causes a release of neurotoxins from one side and leads to CBF reduction on another side. In the Aβ-dependent pathway (green), the BBB breakdown impairs the clearance of Aβ and APP (amyloid precursor protein), leading to the aggregation of Aβ in the brain. The accumulated Aβ and vascular hypoperfusion phosphorylate tau, leading to the formation of NFTs. In addition, the deposited Aβ also cause inflammation in the brain. In conclusion, both factors and pathways cause neurodegeneration leading to dementia (AD) (Iadecola and Davisson, 2008; Jack, 2010; Winkler et al., 2011; Sagare et al., 2013; Edwards et al., 2019) as shown (Figure 4).

#### Phenotypes of BBB Breakdown in AD

In AD patients, the BBB is shown as leakages in brain vasculature, the perivascular aggregation of fibrinogen, albumin, thrombin, and immunoglobulin (IgG), the loss of TJs, and the degeneration of ECs and pericytes (Nelson et al., 2016). Furthermore, identical phenotypes were also observed in *Apoe*^−/−^ mice due to BBB impairment (Nishitsuji et al., 2011; Bell et al., 2012; Hammer et al., 2014; Soto et al., 2015; Castillo-Gomez et al., 2016; Di Cataldo et al., 2016), indicating that ApoE is vital for maintaining BBB integrity.

As pericytes are crucial for maintaining the BBB, any dysfunction in the signaling pathways of pericytes results in the breakdown of BBB, which causes dementia and other neurodegenerative diseases (Sagare et al., 2013; Nikolakopoulou et al., 2019). Brain microvascular endothelial cells (BMEC) secrete platelet-derived growth factor BB (PDGF-BB) and activate PDGFRβ signaling, which is essential for the proliferation, migration, and survival of pericytes (Stratman et al., 2010). An impairment in PDGFRβ signaling leads to pericyte degeneration (Stratman et al., 2010; Nation et al., 2019). According to a previous study, PDGFRβ signaling was decreased in adult *Foxf2* deficient mice, thus resulting in high BBB permeability (Reyahi et al., 2015). Impairment in the BBB was also reported in *Pdgfr*β^+/−^ pericyte-deficient mice, which subsequently caused neuronal degeneration (Bell et al., 2010). In the AD murine model (APP^sw/0^), the deterioration of pericytes results in the dysfunction of the BBB, leading to amyloid β accumulation and tau protein (p-tau) phosphorylation (Sagare et al., 2013). The breakdown of the BBB was also reported in AD patients associated with the reduction in pericytes (Sengillo et al., 2013). Studies also showed that the leakage of the BBB in AD patients starts at the hippocampus, resulting in an increase of soluble PDGFRβ (sPDGFRβ) in the CSF (Montagne et al., 2015; Miners et al., 2019). Additionally, the level of sPDGFRβ in the CSF can be used as a biomarker to predict dementia and other neurodegenerative diseases such as AD (Nation et al., 2019).

Astrocytes are one of the main components of the NVU and are essential for the integrity of the BBB. In an *in vitro* study, it was observed that Sonic hedgehog (Shh) signaling released from astrocytes plays a vital role in the maintenance of BBB integrity by upregulation of *CLDN5* and *OCLN* (Alvarez et al., 2011; Wang et al., 2014). Recently, it was also reported that, in the stroke mouse model, ischemia-induced astrogliosis led to the downregulation of the expression of TJ protein claudin-5 and occludin (Matthes et al., 2021), suggesting that astrocytes have a role in the regulation of TJ proteins. Another recent study reported that, in the tamoxifen-induced astrocyte ablation adult mouse model, the expression of TJ protein ZO-1 was downregulated in vessel regions where astrocyte loss occurred, which may show the role of astrocytes in maintaining the integrity of the BBB in adult brains (Heithoff et al., 2021). A conditional knockout mouse study also showed that the deletion of laminins in astrocytes caused a decline in astrocytic AQP4 (Aquaporin4) expression, thus leading to a loss of TJ in ECs (Yao et al., 2014). In the AD brain, various changes in the morphology of astrocytes have been reported to cause BBB breakdown (Cai Z. et al., 2017). The depolarization of astrocyte endfeet may diminish the integrity of BBB, which was reported in the tg-ArcSwe mouse model of AD(Yang J. et al., 2011). In AD models, researchers also identified several changes in the morphology of astrocytes endfeet near aggregated vascular Aβ (Kimbrough et al., 2015).

A mouse study showed that microglia stimulate TJ protein claudin-5 expression and maintain BBB integrity (Haruwaka et al., 2019). However, the BBB integrity becomes compromised with prolonged inflammation through the changing of the morphology of microglia (Lassman et al., 2012; Haruwaka et al., 2019). In the AD brain, due to the accumulation of Aβ, microglia activate and secret inflammatory cytokines, such as interleukins (IL-1 and IL-6) and tumor necrosis factor (TNF-α, and TNF-β) (Zhou et al., 2012) that cause BBB impairment (Wang et al., 2014). As a result, the trafficking of neutrophils through the BBB becomes elevated due to BBB breakdown (Allen et al., 2012; Wang et al., 2014; Zenaro et al., 2015). Furthermore, it has been observed that, in the tamoxifen-induced astrocyte knockout adult mouse model, the loss of astrocytes causes the activation of microglia (Heithoff et al., 2021); in turn, the activated microglia produce reactive oxygen and reactive nitrogen species (RNS), leading to BBB dysfunction and neurodegeneration (Block, 2008; Sumi et al., 2010).

In the physiological state, perivascular macrophages (PVMs) have a significant role in the maintenance of TJs between ECs. They also decrease vessel leakage, degrade pathogens, and limit inflammation (Lapenna et al., 2018) while contributing to BBB breakdown in the disease state (Boyle et al., 2018). These PVMs are enriched with scavenger receptors, might be involved in the clearance of toxin products from the brain parenchyma (Faraco et al., 2017), and have a diverse role in disease states such as AD (Lapenna et al., 2018). Perivascular macrophages have been shown to phagocytose and alleviate Aβ plaques, and the PVM-deficient mouse model showed an increased aggregation of Aβ42 and cerebral amyloid angiopathy (CAA) related with AD (Yang et al., 2019). Another study showed that PVMs that are deficient in CD36 and Nox2 abrogated the production of ROS and Aβ cerebrovascular impairment compared with wild-type mice (Park et al., 2017).

In addition, perivascular fibroblasts (FBs) express the ECM genes *col1a2* and *col5a1* and are considered to mediate blood vessel integrity. Zebrafish deficient with *col5a1* showed spontaneous hemorrhage in the presence of the additional genetic ablation of the *col1a2* gene, suggesting the role of perivascular FBs in stabilizing vascular integrity (Rajan et al., 2020). Perivascular FBs also express *Lama2, Lamb1*, and *Lamc1*, which encode laminin 211 that interacts with astrocytic dystrophin, resulting in the regulation of AQP4 in astrocytic endfeet. This study suggests that any impairment in perivascular FBs result in the dysregulation of AQP4, which may cause Aβ aggregation and AD as reviewed by Lendahl et al. (2019). Furthermore, it has been reported that alteration in the activity of perivascular FBs also leads to other neurological disorders (Månberg et al., 2021).

#### Mechanisms of BBB Breakdown in AD

Various pathological and aberrant events such as oxidative stress, inflammation, and the ApoE4 genotype cause BBB breakdown associated with AD. Research has shown that, in AD, the activation of the inflammatory and oxidative stress signaling pathways is the primary event that causes BBB disruption (Perry et al., 2002; Candore et al., 2010; Eikelenboom et al., 2012). Cytokines (Pan et al., 2011), Aβ (Gonzalez-Velasquez et al., 2008; Deli et al., 2010; Carrano et al., 2011), LPS (Bannerman and Goldblum, 1999; Verma et al., 2006), and p-tau proteins (Kovac et al., 2009) are the stimulus of inflammation for the activation of inflammatory pathways in BBB ECs. Hence, increases of the pro-inflammatory mediators and ROS/RNS in BBB ECs, astrocytes (Tada et al., 1994), and pericytes (Kovac et al., 2011; Takata et al., 2011) ultimately cause BBB breakdown. Recently we reviewed the role of peripheral inflammation in BBB breakdown (Huang et al., 2021). Glucose transporter protein (GLUT1) is repressed in the endothelium of AD, which causes a decline in the glucose level of the CNS (Mooradian et al., 1997; Winkler et al., 2015). In human AD, LRP1, which is a primary receptor for the clearance of amyloid-β, is downregulated with an increase in oxidative stress (Deane et al., 2004; Donahue et al., 2006; Sagare et al., 2007; Miller et al., 2008; Owen et al., 2010; Halliday et al., 2016); as a result, the transport of Aβ from the brain becomes reduced and leads to amyloid-β accumulation in the brain (Deane et al., 2004, 2008; Storck et al., 2016). In mice, systemic inflammation with LPS has been observed to downregulate both LRP-1 and P-gp efflux transporters and block the Aβ clearance from the brain (Jaeger et al., 2011; Erickson et al., 2012). Furthermore, it has been reported that the expression levels of the receptor for advanced glycosylation end products (RAGE) in both mural cells and brain endothelium were elevated (Deane et al., 2003; Donahue et al., 2006; Miller et al., 2008). The function of the RAGE is to transfer Aβ from blood to the brain (opposite to LRP1), which enhances neuronal inflammation. In AD patients, the RAGE is observed as a significant therapeutic target (Bell et al., 2012). A transgenic mouse with overexpressed APP (amyloid precursor protein) has been reported to show vascular impairment due to the elevation of Aβ40 (Niwa et al., 2000).

Aquaporin-4 (AQP4) is the prime water channel expressed in the CNS and is primarily expressed in astrocytes, thus playing a vital role in normal brain homeostasis and various neurological diseases (Lan et al., 2016b). Specifically, AQP4 facilitates the clearance of Aβ, and alteration in AQP4 expression leads to the accumulation of amyloid-β in the brain (Hoshi et al., 2012; Yang et al., 2012). Furthermore astrocytic AQP4-deficient animals cannot efficiently remove Aβ from the brain (Iliff et al., 2012). A study showed that AQP4 is crucial to regulate fluid flow in the brain interstitial required to maintain the microenvironment for neurons to function properly. The perturbed AQP4 expression has been observed to cause Aβ deposition and inflammation in the human brain, which leads to AD (Rasmussen et al., 2018). In AD patients and animal models, the expression and distribution of AQP4 were altered, leading to amyloid-β accumulation, which plays a vital role in the pathogenesis of AD as reviewed by Yang et al. (2016). Furthermore, it has been observed that, in AD patients, the localization of AQP4 in the perivascular space was reduced and is associated with an increase in neurofibrillary and amyloid-β pathology (Zeppenfeld et al., 2017). In addition, AQP4 facilitates the transport of potassium and calcium ions, which plays an essential role in the pathogenesis of AD as reviewed by Lan et al. (2016a). In AD, the chronic activation of microglia leads to the release of abundant pro-inflammatory cytokines and abolishes phagocytosis, thus causing the deposition of Aβ and neuroinflammation (Krabbe et al., 2013; Heneka et al., 2015) and subsequently producing ROS that causes BBB dysfunction and neurotoxicity (Block, 2008; Sumi et al., 2010). The activated microglia also release IL-1β (a pro-inflammatory cytokine) that amplify the BBB leakage and diminish the ability of the astrocytes to maintain the BBB (Wang et al., 2014). Therefore, AQP4 can be a fascinating therapeutic target for AD and other CNS diseases.

Apolipoprotein E (ApoE) is a protein encoded by the *APOE* gene, located on chromosome 9 and associated with lipid transport. *APOE* consists of three alleles, namely, ε*2*, ε*3*, and ε*4*, translated to ApoE2, ApoE3, and ApoE4 isoforms. The *APOE* isoform distributed as *APOE3* is the most abundant in humans at approximately 77.9%, while *APOE4* and *APOE2* distributions are 13.7 and 8.4%, respectively (Farrer et al., 1997). In the CNS, astrocytes produce ApoE, whereas, in peripheral tissue, ApoE production occurs in the liver (Liu et al., 2013).

**Figure 5:**
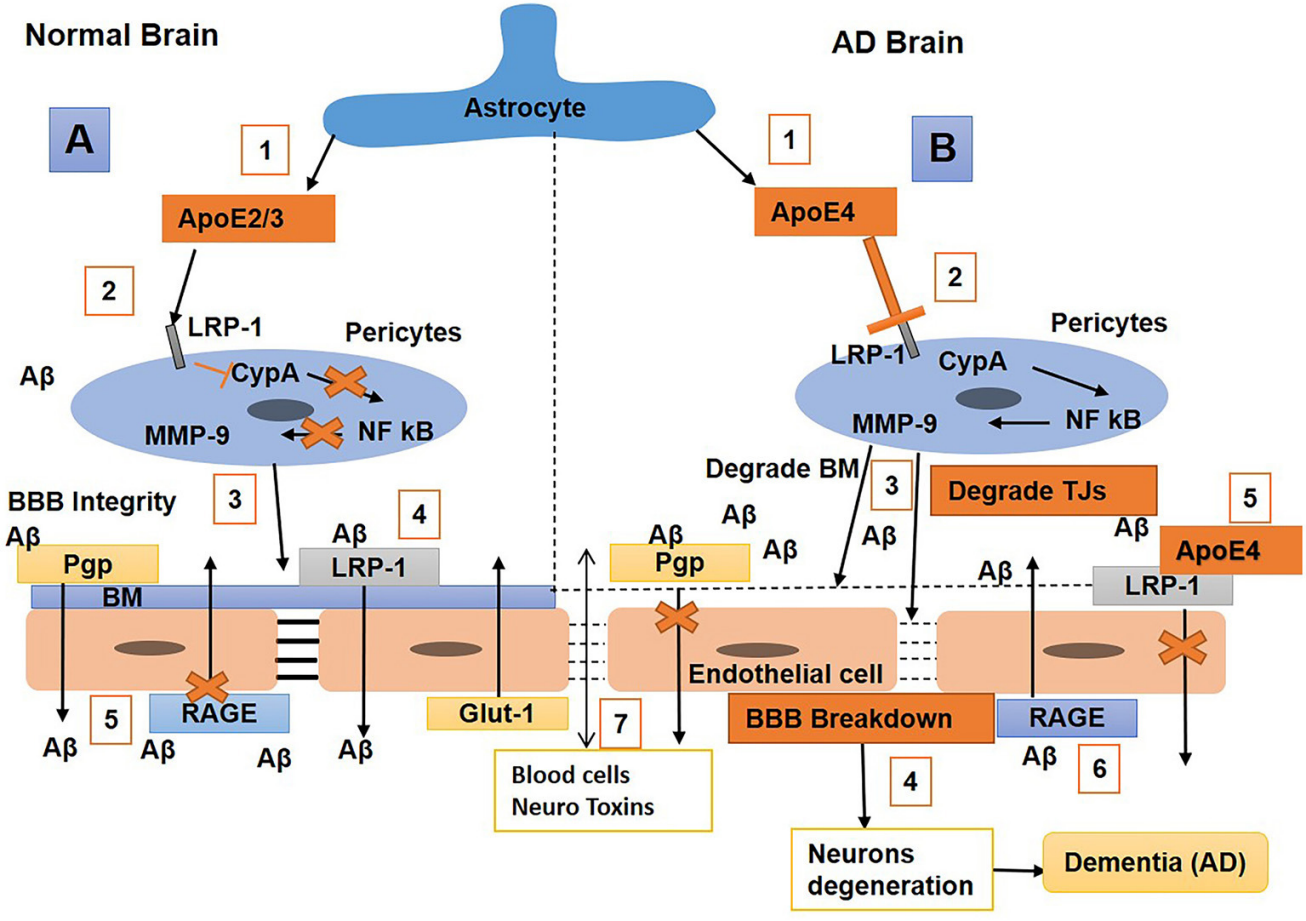
Schematic diagram shows the mechanisms of blood–brain barrier (BBB) breakdown in a normal brain and one with Alzheimer's disease (AD). **(A) Normal brain** (1) Astrocytes release ApoE2/3, (2) bind with the low-density lipoprotein receptor-related protein-1 (LRP-1) on pericytes and repress CypA-NFkB, which, in turn, stops matrix metalloproteinase 9 (MMP9) secretion in pericytes; (3) hence, maintaining BM and BBB integrity, with (4) LRP-1 and P-gp on endothelial cells (ECs) also helping in amyloid-beta (Aβ) clearance. (5) Receptor for advanced glycosylation end products (RAGE) expression is repressed to stop the transport of Aβ into the brain. **(B) AD brain** (1) Astrocytes secret ApoE4, (2) weakly interact with LRP-1 on pericytes which activates the cyclophilin-A nuclear factor kβ matrix metalloproteinase 9 (CypA-NFkB-MMP9 pathways), (3) and result in BM and tight junctions (TJs) degradation leading to BBB breakdown, (4) associated with neurodegeneration and dementia. (5) ApoE4 also weakly interacts with LRP-1 on ECs that cannot significantly clear Aβ from the brain; hence, Aβ accumulates in the brain, causing neuronal damage. (6) Also, RAGE expression is upregulated, which promotes the transport of Aβ from blood to brain. (7) Blood cells and neurotoxins diffuse into the brain and cause neuronal loss and dementia.

Studies reported that ApoE plays an essential role in maintaining BBB integrity (Nishitsuji et al., 2011). An *in vivo* study showed that ApoE2/3 induces BBB integrity by interacting with LRP-1 on pericytes to block the cyclophilin-A nuclear factor kβ matrix metalloproteinase 9 (CypA-NF-kβ-MMP-9) pathway, thus resulting in the inhibition of MMPs (Bell et al., 2012). Researchers also observed that the *APOE4* isoform is a major risk factor for AD, and that the binding of Aβ with apoE4 shifts fast clearance of soluble Aβ40/42 from LRP1 to VLDLR; hence, Aβ-apoE4 complexes at the BBB are cleared with a slower rate than LRP1 (Deane et al., 2008; Tachibana et al., 2019). The expression of *APOE4* causes a reduction in BBB integrity by promoting pericyte degeneration in AD (Bell et al., 2012), which is correlated with high BBB permeability to IgG and fibrin (Halliday et al., 2016). In a transgenic mouse study, the mice that had *Apoe* replaced with human *APOE* (*TR-APOE*) showed astrocytes that secreted ApoE4 blocks pericytic LRP-1, resulting in the activation of the proinflammatory CypA-NF-kB MMP9 pathway, BBB disruption, and brain hemorrhage through the enzymatic breakdown of the TJ and basement membrane (Nishitsuji et al., 2011; Bell et al., 2012). A study showed that an LRP1 endothelial knockout caused the activation of the CypA–MMP9 pathway in the endothelium, which led to damage to TJs and BBB breakdown (Nikolakopoulou et al., 2021). In *TR-APOE4* mice, the repression of Glut1 and upregulation of RAGE expression were also observed compared with *TR-APOE3* or *TR-APOE2* (Alata et al., 2015). It has been reported that humans carrying *APOE4* are more prone to breakdown in the BBB and loss of pericytes than non-*APOE4* carriers (Hultman et al., 2013; Zonneveld et al., 2014; Halliday et al., 2016). Furthermore, CypA and MMP-9 levels increase in *APOE4* carriers, leading to the elevation of IgG and fibrinogen leakages (Halliday et al., 2016). Overall, these results suggest that ApoE2/3 represses inflammation by interacting with pericyte LRP-1, subsequently inducing BBB integrity. In contrast, the ApoE4 might have BBB impairment properties or cause a higher risk of BBB breakdown. The repression of ApoE4 or inhibition of the CypA–MMP9 pathway in humans with AD might be an exciting topic in the future for the reduction the neurodegenerative process (Figure 5).

### BBB Breakdown in VaD

Vascular dementia is a neurodegenerative disease caused by reduced CBF to the brain resulting in cognitive dysfunction. After AD, VaD is considered the second most common dementia, accounting for ~15–30% of all dementia (Sloane et al., 2002; Abou-Saleh et al., 2011; Gorelick et al., 2011; Goodman et al., 2017).

#### Pathophysiology of VaD

Chronic hypoperfusion and thrombosis are the main factors in VaD that cause reduced CBF and promote oxidative stress, hypoxia, and inflammatory molecule expression (cytokines/chemokines). These chronic events cause damage to the periventricular WM, basal ganglia, and hippocampus. Cerebrovascular pathology has a significant contribution to the pathogenesis of VaD by damaging the brain. Vascular impairments include large vessel atherosclerosis (AS), small vessel AS, and CAA. These cerebrovascular pathologies cause microinfarcts in gray matter, WM lesions, and microbleeds (Thal et al., 2012). These vascular abnormalities can occur throughout the brain, resulting in VaD (Grinberg and Heinsen, 2010).

#### Phenotypes of BBB Breakdown in VaD

**Figure 6:**
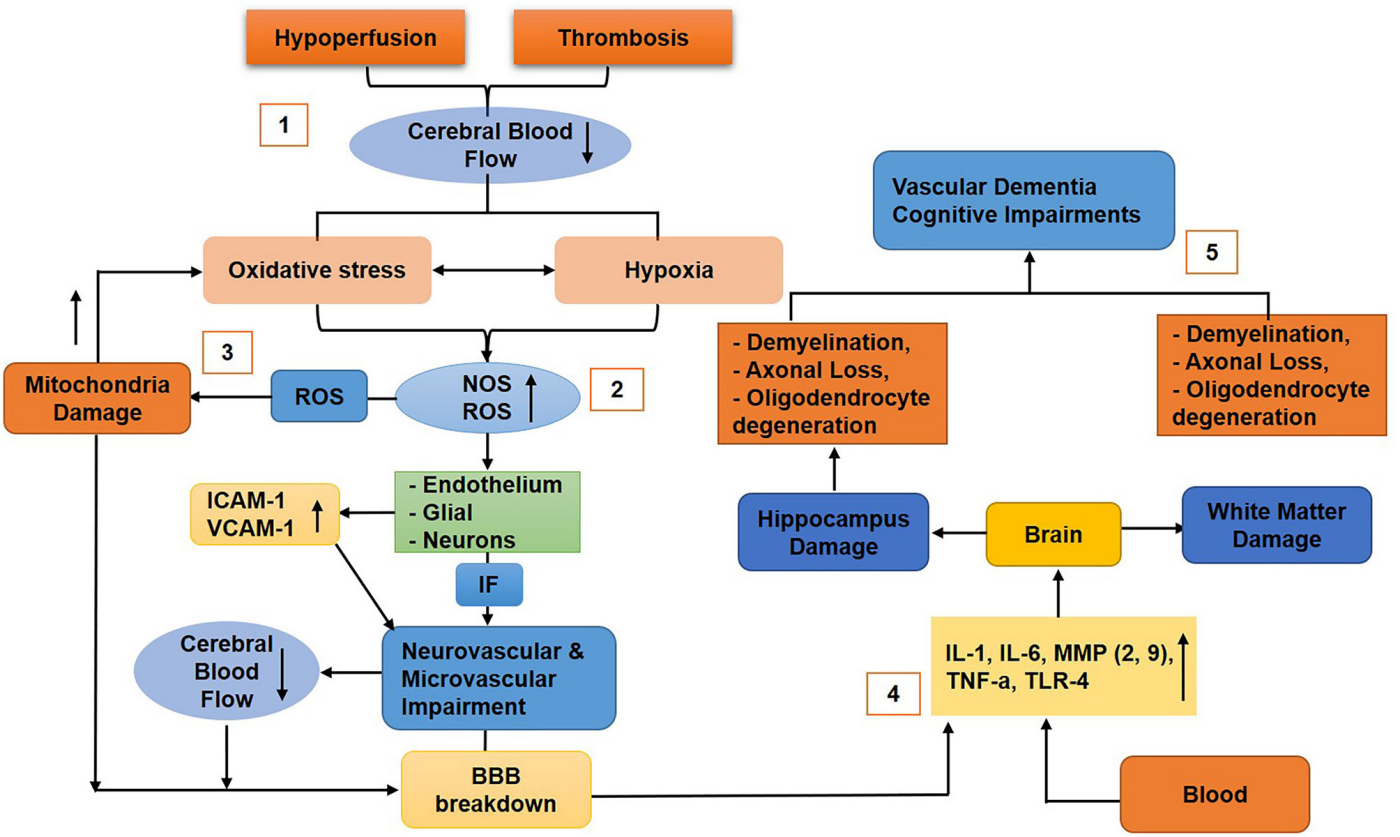
Diagram that shows the molecular mechanism of blood–brain barrier (BBB) breakdown in vascular dementia. (1) Hypoperfusion and thrombosis cause reduced cerebral blood flow (CBF), thus generating oxidative stress and hypoxia (2), which upregulate nitric oxide and reactive oxygen species (ROS) that stimulate the endothelial (ICAM-1, VCAM-1 upregulated), glial, and neuronal cells to release inflammatory factors that cause neurovascular unit (NVU) impairment and reduce CBF and BBB disruption. (3) ROS damage mitochondria of the BBB cells that will further upregulate oxidative stress, resulting in BBB impairments. (4) Cytokines, chemokines, toxins, and other inflammatory molecules infiltrate the brain and cause damage to the hippocampus and white matter (5) associated with neuronal loss, vascular dementia, and cognitive impairments.

Hypertension is one of the factors that cause BBB breakdown in VaD with the accumulation of perivascular collagen in the hippocampus and WM lesions (Verhaaren et al., 2013). Toxic molecules or high blood pressure cause damage to the BBB endothelium. Hypertension also causes a reduction in the integrity of ECs and pericytes, astrocytes endfeet swelling, and retraction from the vessel wall, which results in BBB breakdown and subsequently leading to a reduction in CBF (Wardlaw et al., 2003). Studies reported that acute ischemia induces BBB permeability by the secretion of ROS (Abboud et al., 2007; Simpkins et al., 2016). A study also showed that, during vascular pathology, chronic hypoperfusion causes BBB disruption in WM lesions (Tomimoto et al., 1996). Another study showed that BBB disruption due to the degeneration of pericytes results in the disruption of WM circulation, deposition of fibrinogen, and reduction of CBF that further induces damage to the myelin, axons, and oligodendrocytes (Montagne et al., 2018) (Figure 6). Furthermore, animal experiments showed that chronic cerebral hypoperfusion (CCH) increases BBB leakage to intravenously injected horseradish peroxidase (HRP) in the corpus callosum. In animals, perivascular collagen was also accumulated in the corpus callosum associated with WM lesion formation and elevated BBB permeability (Ueno et al., 2002). In VaD, PVMs have been reported to induce oxidative stress leading to hypertension (Yang et al., 2019).

#### Mechanisms of BBB Breakdown in VaD

Hypoxia upregulates oxidative stress, which produces NO, ROS, and free radicals (Li et al., 2013; Ma et al., 2013; Zhang et al., 2014). In addition, oxidative stress disrupts the ratio of antioxidants, NO, and ROS and causes damage to the endothelial, glial, and neuronal cells, resulting in the impairment of the NVU, BBB disruption, and mediation of a reduction in CBF (Liu and Zhang, 2012). In particular, ROS can further lead to mitochondrial dysfunction resulting in cerebral hypoxia that induces oxidative stress (Zhang et al., 2014). Cerebral vascular hypoxia produces inflammatory molecules that cause apoptosis and impairments in the function of microvessels. The cytokines/chemokines cause damage to the endothelium, glial, and neurons cells and, hence, enhance BBB permeability (Gill et al., 2010). The inflammatory molecules such as IL-1, IL-6, MMPs (MMP-2, MMP-9), TNFα, and TLR4 (toll-like receptor 4) infiltrate the brain (Li and Lai, 2007; Gill et al., 2010; Candelario-Jalil et al., 2011; Reuter et al., 2015), cause demyelination, and damage the axons and oligodendrocytes associated with the hippocampus and WM lesions (Chen et al., 2011).

Damage to oligodendrocytes represses remyelination (Ihara et al., 2010), and demyelination retains the transmission of neural signals, thus resulting in cognitive impairment. Overall, hypoxia, oxidative stress, and inflammation cause defects in neurogenesis, impairment in the proliferation of neuronal progenitor cell, synaptic plasticity, and reduced spine density in the hippocampus, thus resulting in cognitive impairment (Stranahan et al., 2008; Park et al., 2010) (Figure 6). Furthermore, several studies reported that CCH causes AD and VaD (Du et al., 2017). It has also been reported that intercellular adhesion molecule 1 (ICAM-1) and vascular adhesion molecule 1 (VCAM-1) were significantly upregulated in the vascular ECs of the CCH animal model associated with cognitive impairment (Won et al., 2013; Khan et al., 2015).

### Overlap Between Alzheimer's and Vascular Dementia

As discussed above and in other studies, significant clinical heterogeneity has been shown between AD and VaD (Sachdev et al., 2014; Chui and Ramirez-Gomez, 2015); however, recent studies reported that these two diseases co-occur in what is called mixed dementia (Emrani et al., 2020). In mixed dementia, vascular pathology not only mediates AD progression, but the pathology of AD also potentiates vascular impairments, suggesting that pure AD or VaD rarely occur (Emrani et al., 2020). In addition, another study reported that, in aged individuals, there is genetic overlap between vascular dysfunction and AD that is primarily associated with apolipoprotein E (Lin et al., 2019).

Community and epidemiological studies reported the mixed neuropathology that is quite common in both AD and VaD (Schneider et al., 2009; Wharton et al., 2011). The clinical study observed that only 9% of 1,000 patients with cognitive impairments have pure AD pathology; however, AD pathology is mainly associated with vascular dysfunction or other neurodegenerative diseases (Boyle et al., 2018). Another clinical study examined 63 patients with mild cognitive impairment (MCI), in which only 28% were reported as pure AD and approximately 24% were diagnosed with mixed dementia (AD and VaD) (Silbert et al., 2012). Researchers observed that frontal lobe lesions and vascular pathology, e.g., white matter hyperintensities (WMH), are associated with neuropsychiatric symptoms and are common in both AD and VaD (Anor et al., 2017). Alzheimer's disease and VaD share many similar clinical pathologies that lead to cognitive impairment and neuropsychiatric symptoms associated with behavioral alterations (Kalaria, 2002) as shown in Table 2. Hence, these studies suggest that there might be considerable overlaps between AD and VaD, and comprehensive studies should be considered to understand the pathophysiology of dementia instead of segregating AD from VaD.

**Table 2:** Pathologies associated to both Alzheimer's disease and vascular dementia.

**Clinical pathologies**	**Alzheimer's disease (%)**	**Vascular dementia (%)**
Cerebral amyloid angiopathy	98	30
Microvascular degeneration	100	30
Total infarctions	36	100
Micro-infarcts	31	65
Intracerebral hemorrhage	7	15
White matter lesions	35	70
Loss of cholinergic neurones	70	40
Cardiovascular disease	77	60

## Biomarkers Associated With BBB Breakdown

Various imaging techniques and other methods are currently being used to identify biomarkers associated with BBB breakdown in different neurological disorders, which are helpful in healthcare decisions. However, during the acute phase of BBB disruption, some of the clinical care places may lack the facilities to perform MRIs; hence, the detection of peripheral blood biomarkers is the best approach to identifying the status of BBB.

Studies showed that, while the blood/CSF albumin ratio can be used as a biomarker to detect BBB permeability, it cannot distinguish BBB and blood-CSF permeability nor locate leakage as reviewed by Farrall and Wardlaw (2009). Hence, nowadays, the dynamic contrast-enhanced MRI (DCE-MRI) technique is used to directly identify and localize these elusive permeability values (Raja et al., 2018). A study in healthy, aged individuals using DCE-MRI with a gadolinium-based contrast agent injected intravenously identified that BBB leakage was high and localized in the brain regions most vulnerable to damage from aging (Verheggen et al., 2020). It has been observed that, by using DCE-MRI, the BBB permeability index *Ktrans* was increased in the hippocampus and some of its sub-regions, CA1 and dentate gyrus (DG), but not in CA3. This study showed that, in the hippocampus, the BBB integrity was lost progressively with age. Still, no significant BBB leakage was observed in the cortical and sub-cortical regions (Montagne et al., 2015), suggesting that, in terms of aging, the BBB breakdown starts in the hippocampus. A study using CSF biomarkers and the DCE-MRI technique reported that aged people with prior cognitive impairment had higher BBB permeability than healthy individuals (Nation et al., 2019). These studies suggest that it is possible to detect and localize BBB leakage by using DCE-MRI.

It has been observed that, in epileptic patients, the levels of serum Visinin-like protein 1 (sVILIP-1) and serum caveolin 1 (sCAV-1) are higher, which may be used as biomarkers for the diagnosis of BBB breakdown (Tan et al., 2020). Another protein biomarker is s100β, which is produced by astrocyte endfeet; when the BBB becomes compromised, s100β is immediately released into the peripheral blood (Kadry et al., 2020). Furthermore, a study identified that the expression levels of A-kinase anchoring protein 7 (AKAP7) were high in the peripheral blood (lymphocyte), and thus might be considered to identify BBB breakdown during ischemic stroke or post-stroke (O'Connell et al., 2017). Neuron-specific enolase (NSE) and GFAP are also promising biomarkers that can be detected in the CSF to identify BBB breakdown (Kadry et al., 2020). A study reported that the elevated level of sPDGFRβ is associated with damage to the pericytes and BBB disruption leading to a decline in cognition (Sweeney et al., 2020). Soluble PDGFRβ as a biomarker was also observed in VaD (Iadecola, 2017; Sweeney et al., 2019a) and various other neurological diseases (Sweeney et al., 2018a, 2019b). Soluble cell adhesion molecules (CAMS), zonulin, and soluble 4-1BBL (transmembrane protein receptor) have also been identified to be associated with BBB damage. PECAM-1, P-selectin, and E-selectin are soluble adhesion molecules reported to be upregulated in individuals with compromised BBB and can be used as biomarkers for BBB breakdown (D'Ambrosio et al., 2015). Increased leakage of gadolinium (DCE-MRI; K*trans*), microbleeds (T2^*^-weighted and SWI-MRI), reduced glucose transport (FDG-PET), diminished P-glycoprotein 1 function (verapamil-PET), and CNS leukocyte infiltration (MMP inhibitor-PET) are some of the techniques that can be used to identify biomarkers associated with BBB damages in various CNS diseases (Sweeney et al., 2018b).

## Conclusions and Future Directions

The BBB consists of a set of physiological properties that tightly regulate the normal microenvironment essential for proper neuronal activities. Any impairment in these properties either at the cellular or molecular level causes BBB breakdown. Aging is one of the factors that contribute to BBB disruption. During aging, the various physiological properties of the BBB are impaired, leading to BBB dysfunction. The neurotoxins infiltrating the brain can also cause cognitive impairments and neurodegeneration. Furthermore, BBB breakdown also contributes to dementia that includes ADs and VaD. In AD with disruption of BBB, Aβ and NFT of p-tau accumulate in the blood vessel, causing further inflammation in the NVU that, in turn, induces the release inflammatory factors to degenerate neurons associated with a decline in cognition. Another factor that degrades the integrity of BBB associated with AD is *APOE4*. In dementia, VaD accounts for the most cases next to AD caused by BBB breakdown. In VaD, the CBF is reduced and inflammatory molecules infiltrate the brain due to BBB impairment, subsequently causing neuronal loss and, thus, cognitive impairment. Hence, BBB breakdown can be used as a novel biomarker to study various neurological impairments such as AD, VaD, and other associated declines in cognition.

Recently, RepSox was identified to inhibit TGF-B, VEGFA, and inflammatory gene networks (Roudnicky et al., 2020). Furthermore, RepSox significantly elevated BBB resistance, induced TJs and transporters, reduced paracellular permeability by activating Notch and Wnt pathways, and, thus, might be used as an emerging BBB therapeutics to treat neurological diseases such as AD (Roudnicky et al., 2020). In addition, secreted protein acidic and rich in cysteine (SPARC) was identified to decrease transendothelial electrical resistance (TEER) and TJ proteins (ZO-1, OCLN) and increase paracellular permeability by regulating the tyrosine kinase pathway (Alkabie et al., 2016). Hence, the SPARC–collagen binding domain might be a potential therapeutic target to treat AD (Pilozzi et al., 2020). Furthermore, SPARC/Hevin normalization may also be considered as a novel therapeutic target for the modulation of AD progression (Strunz et al., 2019).

Although researchers have reported the contributions of BBB disruption to the pathogenesis of cognitive impairment associated with normal aging and dementia, more research is needed to elucidate the precisely causing factors and the cellular and molecular mechanisms of BBB maintenance, breakdown, and repair correlated with neurodegeneration and cognition decline. In the future, how aging and dementia affect BBB function in health and disease state, thus leading to neurodegeneration and cognitive impairment, should be explored in living organisms. Clinical research pertaining to this will boost our knowledge and help us better understand the association between BBB breakdown and cognitive decline. Such studies pave the way for the use of the BBB as a novel biomarker and therapeutic target to treat dementia and other neurological diseases associated with cognitive impairment. Furthermore, these studies suggest that amelioration in the cerebrovascular pathways (particularly BBB breakdown) can alleviate neurodegeneration in dementia (particularly AD) associated with cognitive impairment. Hence, the characterization of the cellular and molecular constituents of the cerebrovascular systems that contribute to the pathophysiology of dementia will provide a systematic methodology of dementia diagnosis. More profound knowledge of the vascular system will also help design emerging efficient strategies that can be used for the therapeutic interventions of cognitive impairment and dementia.

## Author Contributions

BH drafted the manuscript and made the figures and table. CF and JC discussed and revised the manuscript. All authors contributed to the article and approved the final manuscript.

## Conflict of Interest

The authors declare that the research was conducted in the absence of any commercial or financial relationships that could be construed as a potential conflict of interest.

## Publisher's Note

All claims expressed in this article are solely those of the authors and do not necessarily represent those of their affiliated organizations, or those of the publisher, the editors and the reviewers. Any product that may be evaluated in this article, or claim that may be made by its manufacturer, is not guaranteed or endorsed by the publisher.
